# Acute respiratory distress syndrome due to sepsis caused by *Bacteroides ovatus* after acute appendicectomy

**DOI:** 10.1186/s40792-022-01475-w

**Published:** 2022-06-20

**Authors:** Yoshinobu Fuse, Hironori Ohdaira, Teppei Kamada, Junji Takahashi, Keigo Nakashima, Yuichi Nakaseko, Norihiko Suzuki, Masashi Yoshida, Shinya Okada, Yutaka Suzuki

**Affiliations:** 1 Department of Surgery, International University of Health and Welfare Hospital, 537-3, Iguchi, Nasushiobara City, Tochigi 329-2763 Japan; 2 Department of Pathology, International University of Health and Welfare Hospital, 537-3, Iguchi, Nasushiobara City, Tochigi 329-2763 Japan

**Keywords:** Appendectomy, Acute respiratory distress syndrome, Sepsis

## Abstract

**Background:**

Appendicectomy is generally a minimally invasive surgery, after which postoperative complications such as acute respiratory distress syndrome (ARDS) are rare. We describe a case of ARDS due to sepsis caused by *Bacteroides ovatus* after appendicectomy.

**Case presentation:**

A man in his 60 s presented to our hospital with a chief complaint of right lower quadrant abdominal pain. He was diagnosed with acute appendicitis and underwent emergency laparoscopic appendicectomy. Cefmetazole was administered as a perioperative antibacterial drug. Postoperatively, the abdominal findings improved. However, on postoperative day three, bloody sputum and respiratory distress were observed. We performed thoracoabdominal computed tomography (CT) and observed bilateral pleural effusion and mottled frosted glass shadows extending to both lung fields. ARDS was diagnosed. We treated the patient with steroids and sivelestat sodium and switched the antibacterial drug to meropenem. The patient’s general condition improved. After the patient was treated, *Bacteroides ovatus* was isolated from preoperative blood culture, which was resistant to cefmetazole.

**Conclusions:**

We encountered a case in which ARDS due to sepsis was caused by *Bacteroides ovatus* after acute appendicectomy. Blood culture to isolate the causative organism and determine its antimicrobial sensitivity after commencement of empiric antibiotics is important even in common diseases, such as acute appendicitis.

## Background

Acute respiratory distress syndrome (ARDS) is an acute respiratory failure characterized by pulmonary edema due to increased permeability of the alveolar septum associated with severe inflammation. It develops secondary to various underlying diseases and trauma, such as sepsis and severe pneumonia [1].

Acute appendicitis is common among emergency room patients and often cured by surgery or treatment with an appropriate antibacterial drug. Surgical complications after appendicectomy include bleeding, intra-abdominal abscess, fecal fistula, and intestinal obstruction [2]. Appendicectomy is generally a minimally invasive surgery. Moreover, ARDS and sepsis as postoperative complications of appendicectomy are rare.

We describe a case of ARDS due to sepsis caused by *Bacteroides ovatus* after appendicectomy for non-perforated acute appendicitis in a man in his 60 s with no remarkable past medical history.

## Case presentation

A man in his 60 s was admitted to our hospital with a chief complaint of right lower quadrant abdominal pain.

**Fig. 1 a:**
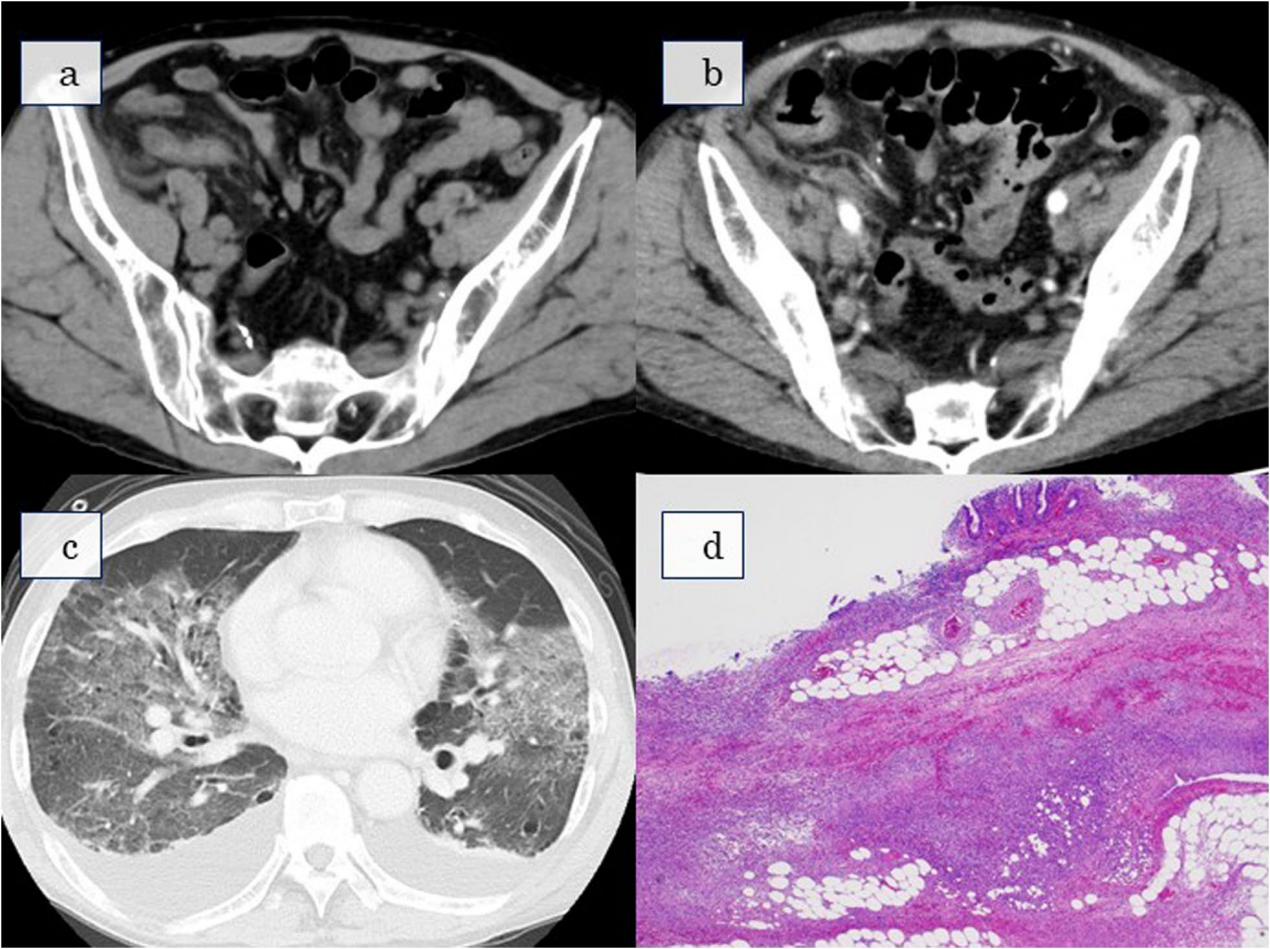
Abdominal CT at initial examination: CT reveals an enlarged appendix in the right lower abdomen. **b** Abdominal CT on postoperative day 3: there is no fluid accumulation suspicious of leakage at the edge of the dissection. **c** Chest CT on postoperative day 3: CT reveals bilateral pleural effusion and extensive mottled frosted glass shadows in the bilateral lung fields. **d** Histopathological analysis: the pathological diagnosis of the resected appendix is gangrenous appendicitis. *CT* Computed tomography

We diagnosed him with acute appendicitis based on findings of localized rebound tenderness in the same area and an enlarged appendix on abdominal computed tomography (CT) (Fig. 1a).

We performed an emergency laparoscopic appendicectomy. The appendix was mildly adherent to the surrounding tissues, and there was a slight leakage of pus during adhesiolysis, which was aspirated and washed out. The appendicular root was dissected after double ligation. A drain was placed near the edge of the dissection. The operation time was 99 min, and the amount of blood loss was 2 ml. No cultures from intra-abdominal abscesses were collected during surgery.

We started the patient on cefmetazole 4 g/day as a perioperative antibacterial drug. Postoperatively, fever reduced and the abdominal findings improved. On postoperative day three, bloody sputum, respiratory distress, and poor oxygenation (PaO_2_/FiO_2_ = 50/0.21 = 250) were suddenly observed. Although oxygen was administered immediately, no improvement was noted (PaO2/FiO2: 60/0.32 = 187.5). Therefore, thoracoabdominal CT was performed. There was no fluid accumulation indicative of leakage at the edge of the dissection (Fig. 1b). However, bilateral pleural effusion and extensive mottled frosted glass shadows in the bilateral lung fields (Fig. 1c) led to the diagnosis of ARDS. There was no edema in the extremities, and cardiac function was normal.

We switched the perioperative antibacterial drug to meropenem (3 g/day) and started methylprednisolone and sivelestat sodium for ARDS. Regarding steroid therapy, we followed the clinical trial protocol of Meduri et al. [3]. This protocol comprised a loading dose of 1 mg/kg followed by infusion of 1 mg/kg/day from day 1 to day 14, 0.5 mg/kg/day from day 15 to day 21, 0.25 mg/kg/day from day 22 to day 25, and 0.125 mg/kg/day from day 26 to day 28. Sivelestat sodium was administered at a dose of 4.8 mg/kg for 7 days [4].

The patient's respiratory condition worsened and he had to be placed on ventilatory support on postoperative day nine. Subsequently, *Bacteroides ovatus* was detected in the blood culture collected preoperatively and was found to be insensitive to cefmetazole. The patient responded to meropenem and steroids, and his respiratory condition gradually improved. He was weaned from the ventilator on postoperative day 19 and discharged on postoperative day 38. The pathological diagnosis of the resected appendix was gangrenous appendicitis only (Fig. 1d).

## Discussion

Complications including ARDS and sepsis following appendicectomy are rare, with only three cases reported in the past literature [5, 6], including our own case.

The findings of three reported cases are summarized in Table 1. All patients were men, and two were young (in their 20 s and 30 s). Two of the three patients had no previous history, and all developed ARDS postoperatively. The causes of ARDS differed between the cases. In the case by Ologun et al. [5], the timing of the surgical procedure was late. In the case by Takeuchi et al. [6], the administration of a large amount of crystalloid solution within 6 h after the patient's arrival at the hospital (despite the absence of septic shock), in addition to asthma, may have contributed to ARDS. The most likely reason for ARDS development in our case was that the perioperative antibacterial drug was ineffective against the bacteria that caused the appendicitis.

**Table 1:** Cases of ARDS after surgery for acute appendicitis

Case	Year	Author	Age	sex	PMH	Perforation	sepsis	ARDS	Symptoms	Blood culture
1	2007	Hiroya Takeuchi	30s	M	−	−	+	+	Bloody sputum respiratory discomfort	Streptococcus constellatus
2	2017	Gabriel O Ologun	25	M	asthma	+	−	+	Respiratory discomfort	Unknow
3	2021	Our case	60s	M	−	−	+	+	Bloody sputum respiratory discomfort	Bacteroides Ovatus

The most common causative organism of appendicitis is *Escherichia coli*, followed by *Enterococcus*. Other causative organisms are *Pseudomonas*, *Klebsiella*, and *Bacteroides*. In a review article by Douglas et al., *Bacteroides* was the causative organism in approximately 10% of 2388 patients with appendicitis [2]. In addition, it has been reported that the proportion of *Bacteroides* as the causative organism of appendicitis increases in cases of advanced appendiceal inflammation, such as gangrenous or perforated inflammation [8].

In the case of ARDS caused by pneumonia, *Streptococcus pneumoniae*, *Staphylococcus aureus*, and *Pseudomonas aeruginosa* are the typical causative organisms [9]. However, the causative organisms of ARDS caused by sepsis other than pneumonia, including after abdominal surgery, have not been clarified. In a study by Dickson et al., *Bacteroides* was the most common bacterium in the bronchoalveolar lavage fluid inpatients with ARDS triggered by sepsis, which suggests that sepsis caused by *Bacteroides* may induce ARDS [10].

In this case, the patient had sepsis due to acute appendicitis, and *Bacteroides ovatus* was detected in blood culture. Although cefmetazole was administered to target anaerobic bacteria, the susceptibility test showed resistance. We suggest that *Bacteroides ovatus* that were present in the bloodstream preoperatively continued to grow postoperatively and might have caused ARDS. We switched the antibacterial drug to meropenem and treated the patient with steroids and sivelestat sodium for ARDS, which improved the patient’s general condition. If a broad-spectrum antimicrobial agent had been used from the beginning, a serious course, as in the present case, could have been avoided.

However, due to bacterial resistance, routine use of broad-spectrum antimicrobial agents for appendicitis is not advisable. It is also quite possible that even if surgery had not been performed, the causative organisms would not have been susceptible to antibiotics and the patient would have developed ARDS as well.

We can compare this present case with a previously reported one [5], in which the patient developed septic shock during antibiotic treatment and underwent emergency surgery. This suggests that the causative organism of appendicitis was resistant to the antibacterial drug (as was the case with our patient), which may have been the cause of septic shock and postoperative development of ARDS. After the onset of ARDS, the patient in the case report by Ologun et al. [5] was treated by changing the antibacterial drug and administering steroids and sivelestat sodium as ARDS treatment, as in our case.

## Conclusions

In this case report, we encountered a case of postoperative sepsis due to *Bacteroides ovatus* followed by ARDS in a patient with non-perforated appendicitis. It is important to recognize that even acute appendicitis could lead to severe outcomes such as sepsis and ARDS if the appropriate antibacterial drugs are not used. We should pay attention to worsening of inflammatory reactions that do not correlate with abdominal findings as well as appearance of bloody sputum and respiratory distress. It is also important to identify the causative organism of appendicitis through cultures of blood and pus from the appendix and to reevaluate the antimicrobial drugs being administered after identification.

## Data Availability

Data sharing is not applicable to this article as no data sets were generated or analyzed during the current study.

## Abbreviations

CT: Computed tomography
ARDS: Acute respiratory distress syndrome
